# Development and performance evaluation of a recombinase polymerase amplification assay for the rapid detection of group B streptococcus

**DOI:** 10.1186/s12866-016-0836-y

**Published:** 2016-09-22

**Authors:** Christina Clarke, Louise O’Connor, Heather Carré-Skinner, Olaf Piepenburg, Terry J. Smith

**Affiliations:** 1 Molecular Diagnostics Research Group, School of Natural Sciences, National Centre for Biomedical Engineering Science (NCBES), National University of Ireland, Galway, Ireland; 2 TwistDx Limited, Cambridge, UK

**Keywords:** Group B Streptococcus, GBS, Neonatal infection, Recombinase Polymerase Amplification, Point-of-care, Near-patient tests, Specificity, Limit of detection, Labour, Neonates

## Abstract

**Background:**

Despite the implementation of prevention guidelines, group B Streptococcal (GBS) infection remains a leading cause of sepsis, pneumonia, and meningitis, resulting in significant neonatal morbidity and mortality. Preventive approaches that identify women at risk of transmitting GBS have reduced the incidence of neonatal GBS disease, and dramatically decreased the associated mortality rates. However, there is an on-going requirement for a near-patient diagnostic test for GBS that can be carried out at the time of delivery, ideally in the labour ward setting, particularly for women of unknown GBS colonisation status at the time of delivery.

**Methods:**

In this study, a Recombinase Polymerase Amplification (RPA) assay was developed and performance evaluated for the detection of group B Streptococcus in vaginal swabs. The assay uses the cAMP factor (cfb) gene of GBS as the target gene. The analytical performance of the assay was evaluated by testing a panel of GBS reference strains and clinical isolates, and non-GBS organisms. The limit of detection was determined and the clinical performance was evaluated by testing 124 vaginal swabs from women with both GBS positive and negative status.

**Results:**

Based on specificity testing carried out the assay was shown to be specific for the target of interest. The limit of detection of the assay was shown to be between six and 12 genome copies and was comparable to that of a real-time PCR assay, both achieving a limit of detection below 12.5 genome copies. The performance of both assays when applied to clinical samples was identical.

**Conclusion:**

A specific, sensitive RPA assay for GBS was developed. The performance of the assay for testing of clinical samples is within the acceptable range.

**Electronic supplementary material:**

The online version of this article (doi:10.1186/s12866-016-0836-y) contains supplementary material, which is available to authorized users.

## Background

*Streptococcus agalactiae*, or Group B Streptococcus infection, emerged in the 1970s as the leading cause of infectious disease in infants, and remains one of the leading causes of neonatal sepsis and pneumonia, sometimes leading to meningitis [1]. Vertical transmission to the infant during birth accounts for 75 % of GBS colonisation of neonates [2], leading to either early onset (less than 7 days) or late onset GBS disease (longer than 7 days but less than 3 months). Early onset GBS infection often presents by the twelfth hour of life and up to 89 % of cases are diagnosed in the first day of life [3]. As a result of significant improvements in diagnosis and disease prevention, the rate of GBS infections, both early and late onset, has significantly reduced, and the mortality rate in neonates has dropped from 50 % to between 4 and 6 %, though for infected preterm infants it can be significantly higher [4, 5]. GBS infection in neonates, particularly if it leads to meningitis, can have significant consequences that can lead to lifelong disabling conditions, including vision impairment, hearing loss and intellectual disabilities [6].

The genital tracts of approximately 25 % of pregnant women are colonised with GBS [7]. This colonisation usually does not become pathogenic to the woman and only becomes problematic in the perinatal setting. Early onset GBS infection can be transmitted to the neonate during birth as it travels through the birth canal of a colonised woman and can subsequently cause infection in the neonate [8]. In some cases it can also be transmitted by ascending infection to the foetus if there is premature rupture of the membranes, puerperal endometritis or if the woman develops chorioamnionitis [8], though, these conditions are relatively rare. It is interesting to note that GBS was a leading cause of fatal puerperal sepsis prior to the introduction of Penicillin [9]. Less is known about the transmission and pathogenesis of GBS in late onset disease. In one study, 64 % of infants with late onset disease were born to GBS positive mothers and the other 36 % are thought to be made up of cases of environmentally acquired GBS [10]. Intrapartum chemoprophylaxis seems to have little effect on late onset disease attack rates.

There are two main schools of thought on the prevention of invasive early onset GBS infection. Some organisations, for example, The Royal College of Obstetricians and Gynaecologists (RCOG) recommends risk-based screening of pregnant women, while others, including the Centres for Disease Control and Prevention (CDC) recommend universal screening of pregnant women. The RCOG do not recommend routine screening for the identification of GBS, which is incidentally found when testing for other infections such as urinary tract infection, or when testing a vaginal swab from a woman with suspected vaginitis [3]. The current CDC guidelines for GBS screening recommend that pregnant women have both rectal and lower vaginal swabs screened using culture or nucleic acid testing. Typical turnaround times for the current gold standard method for GBS detection involving culture from swabs, are between 24 and 72 h depending on the method employed in the laboratory where the testing is to be done and additional time may be required for antimicrobial susceptibility testing. The CDC, in their 2010 GBS report, state that a rapid molecular test to determine GBS colonisation status would be beneficial in the perinatal setting, for women with an unknown GBS colonisation status, to reduce the likelihood of invasive GBS infection of the neonate, providing the assay is sufficiently sensitive and specific [1].

Recombinase Polymerase Amplification (RPA), a rapid, highly sensitive and specific *in vitro* isothermal nucleic acid amplification technology, offers an ideal approach to GBS screening in near-patient settings. RPA-based tests could be used both at the CDC recommended 35 to 37 week gestation screen as well as for screening women who are in labour with an unknown GBS status (e.g. women in preterm labour that have not yet been screened or women who did not seek antenatal care). RPA utilises a number of enzymes, including recombinases and strand-displacing DNA polymerases, to perform DNA or RNA amplification [11]. RPA results are generated very rapidly, typically in under 15 min, and often in under 10, and RPA is able to detect even very few copies of DNA [11]. A recently reported RPA assay for GBS had a limit of detection of 20 genome copies, with positive results available within 8 min from the start of the reaction [12].

RPA has many features which makes it more attractive than PCR-amplification based tests and ideal for point-of-care NAD testing, such as are required to screen pregnant women for GBS colonisation. It does not require thermal cycling, with an optimum temperature range of 35–40 °C, thus negating the need for complex instrumentation. As a result of the extremely rapid cycling times, results are available much more rapidly than achieved with PCR.

The objective of this study was to develop and optimise an RPA-based nucleic acid diagnostic test for GBS that can be performed in a wide variety of near-patient clinical settings, including labour and delivery wards. The RPA test developed in this study targets the CAMP factor cfb gene, which is specific to GBS, and is therefore, ideal for development of a GBS-specific assay. The analytical performance of the GBS RPA test was optimised and the assay was evaluated using clinical samples, and compared to a previously developed GBS real-time PCR assay [13]. The results from these tests were correlated with the results of microbiological analyses of the samples.

## Methods

### RPA reactions

Each RPA reaction contained 2 μl of DNA in a final reaction volume of 50 μl. Master Mix was prepared by adding 37.5 μl primer/probe mix (Table 1) in rehydration buffer, 9.5 μl PCR Grade H_2_O, 4 μl magnesium acetate (280 mM), 2 μl template DNA per reaction at the required concentration. For specificity testing this concentration was 1 × 10^5^ genome copies of DNA from each isolate per reaction. For limit of detection experiments defined copy numbers ranging from 100 to 0.78 were tested. The kit used in this study was the custom Exo kit from TwistDx. All reagents were provided by TwistDx. All RPA experiments were performed at 40 °C on the Twista® instrument (TwistDx UK) device using a reaction time of 20 min. Incubations included a manual mixing step (5 s tube vortex) at 4 min incubation. For negative or no template controls (NTC) these reactions were prepared as normal substituting the target DNA with an equal volume of molecular grade water.

**Table 1:** RPA primer and probe sequences

Oligo name	Sequence 5’-3’
FP1	tctattggtagtcgtgtagaagccttaaca
RP1	tatcccaaatcccatatcaatatttgcttg
P1	agccttaacagatgtgattgaagcaatcact-t(FAM)-t-dSpacer-t(BHQ-1)-caactcaacattta-SpacerC3

### Real Time PCR reactions

A previously developed real time PCR assay for detection of GBS [13] was used to benchmark the RPA assay. The final reaction (20 μL) contained 2 μl LC FastStart DNA Master Hybprobe (Roche) 5 mM MgCl_2,_ 500nM Forward Primer, 500nM Reverse Primer, 200nM each of a fluorescently labelled hybridisation probe pair (Tib Molbiol Germany), 10 μL PCR grade water and 2 μL DNA, at the required concentration. For specificity testing this concentration was 1 × 10^5^ genome copies of DNA from each isolate. For limit of detection experiments defined copy numbers ranging from 100 to 0.78 were tested. Thermocycling conditions consisted of 95 °C for 10 mins, and 50 cycles of 95 °C for 10 s 50 °C for 15 s and 72 °C for 10 s. Reactions were carried out on the LightCycler® Carousel-Based system (Roche).

### Organisms used for inclusivity and exclusivity testing

*S. agalactiae* isolates used to confirm the inclusivity of the assay are listed in Table 2. Organisms used to confirm exclusivity of the assay are listed in Tables 3 and 4. DNA was extracted manually from cultures using the Qiagen Blood and Tissue kit. DNA was quantified using the Qubit analyser (Invitrogen). 1 × 10^5^ genome copies of DNA from each isolate were tested per reaction. Dilutions of the target DNA for limit of detection studies were prepared in molecular grade water.

**Table 2:** Organisms tested to confirm the inclusivity of the RPA assay

Organism	Source ID	Result
*S. agalactiae*	BCCM 15081	Detected
*S. agalactiae*	BCCM 15082	Detected
*S. agalactiae*	BCCM 15083	Detected
*S. agalactiae*	BCCM 15084	Detected
*S. agalactiae*	BCCM 15085	Detected
*S. agalactiae*	BCCM 15086	Detected
*S. agalactiae*	BCCM 15087	Detected
*S. agalactiae*	BCCM 15090	Detected
*S. agalactiae*	BCCM 15094	Detected
*S. agalactiae*	BCCM 15095	Detected
*S. agalactiae*	ATCC 13813	Detected
*S. agalactiae*	ATCC 12386	Detected
*S. agalactiae*	ATCC 27591	Detected
*S. agalactiae*	ATCC 12973	Detected
*S. agalactiae*	ATCC 31475	Detected
*S. agalactiae*	ATCC 12403	Detected
*S. agalactiae*	ATCC BAA-611D	Detected

*BCCM* Belgian Coordinated Collections of Microorganisms

*ATCC* American Tissue Culture Collection

**Table 3:** Streptococcus genus panel used in exclusivity tests for the RPA GBS assay

Organism	Source ID	Result
*Streptococcus anginosus*	DSMZ 20563	Not Detected
*Streptococcus dysgalactiae subsp. equisimilis*	DSMZ 6176	Not Detected
*Streptococcus gordonii*	DSMZ 6777	Not Detected
*Streptococcus intermedius*	DSMZ 20573	Not Detected
*Streptococcus mitis*	DSMZ 12643	Not Detected
*Streptococcus mutans*	DSMZ 20523	Not Detected
*Streptococcus oralis*	DSMZ 20627	Not Detected
*Streptococcus parasanguinis*	DSMZ 6778	Not Detected
*Streptococcus pneumoniae*	DSMZ 11865	Not Detected
*Streptococcus pneumoniae*	DSMZ 11866	Not Detected
*Streptococcus pyogenes*	DSMZ 20565	Not Detected
*Streptococcus salivarius*	DSMZ 20560	Not Detected

DSMZ German collection of microorganisms and cell culture

**Table 4:** Hit rate analysis for the GBS RPA and real-time PCR assays showing number of replicates of each target input concentration detected

Target input genome copies	No replicates tested	No replicates detected in RPA assay	No replicates detected in PCR assay
100	24	24	24
50	24	24	24
25	24	24	24
12.5	24	24	24
6.25	24	20	24
3.1	24	15	14
1.5	24	14	18
0.8	24	10	9

### Limit of detection of the assay

The limit of detection of the assay was determined by preparing known concentrations of GBS (*S. agalactiae* BCCM 15081) DNA and testing in the RPA assay. Eight replicates of concentrations equivalent to 100, 50, 25, 12.5, 6.25, 3.1, 1.5 and 0.78 genome copies in a 2 μL volume were tested in three independent experiments.

### Testing of clinical samples

One hundred and twenty four vaginal swab samples which had been stored at −20 °C in lysis buffer from the BD GenOhm™ Lysis Kit (Beckton Dickenson USA) were tested. This crude lysate was originally prepared by re-suspending swabs in 1 ml of sample buffer. Of this, 400 μL was transferred into a lysis tube and lysed by mechanical disruption with silica beads according to the manufacturer’s instructions. From this crude lysate 2 μL was added directly to either the RPA reaction or the real-time PCR reaction.

## Results

### Analytical specificity of the RPA-GBS assay

**Fig. 1:**
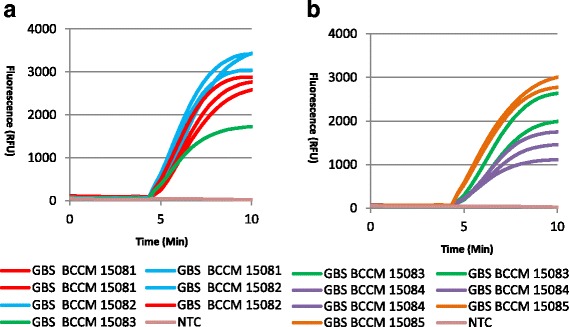
RPA Amplification curves obtained from a panel of GBS isolates tested with the RPA GBS assay. All isolates, listed in Table 1, were tested in triplicate. **a** shows amplification curves obtained for isolates BCCM 15081, 15082, and BCCM15083. **b** shows amplification curves obtained for isolates BCCM 15083, 15084, and BCCM15085 (tested in duplicate)

**Fig. 2:**
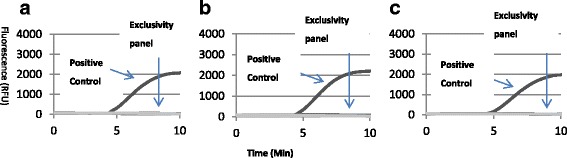
RPA results obtained when a Streptococcus Genus exclusivity panel, listed in Table 2, was tested in the RPA GBS assay. **a**, **b** and **c** show amplification curves for only for the positive control GBS 15081, demonstrating specificity of the assay for GBS

The inclusivity of the assay was determined by testing a panel of GBS isolates. All isolates were tested in triplicate. Table 2 shows the results obtained, while representative RPA amplification curve graphs are shown in Fig. 1. All GBS strains tested were detected. A positive signal (change in fluorescence over background) is detected automatically by the Twista software. The specificity of the assay was further challenged by testing a panel of closely related Streptococcus species. No cross-reaction was observed (Table 3, Fig. 2a, b & c). Finally a panel of organisms associated with the site of infection were tested in duplicate. No cross-reactivity was observed (Additional file 1).

### Limit of detection

The limit of detection (LoD) of the RPA and real-time PCR assays was determined by testing eight replicates of each of the following concentrations, 100, 50, 25, 12.5, 6.25, 3.1, 1.5 and 0.8 genome copies of GBS BCCM15081 in three independent runs. Table 4 shows the hit rate analysis for the combined data from three independent experiments. The LoD of the RPA GBS assay was shown to be between 6.25 and 12.5 genome equivalents, while the LoD of the real-time PCR GBS assay was shown to be in the range of 3.1 and 6.25 genome copies, indicating that the analytical sensitivity of both assays is comparable.

### Clinical performance evaluation

**Fig. 3:**
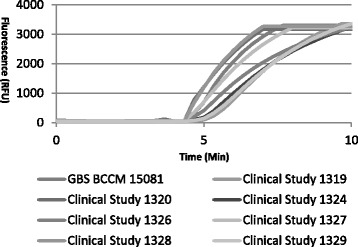
Representative RPA amplification curves obtained for eight of the 124 clinical samples tested in the RPA GBS assay

One hundred and twenty four samples were tested in the RPA-GBS assay. For comparison the same sample set was also tested in the real-time PCR assay. All samples had previously been cultured for GBS. Figure 3 shows representative RPA amplification curves obtained for some of the clinical samples tested. Eighty five samples were positive when tested by RPA and 39 samples were negative (Additional file 2). The same result was obtained when the samples were re-tested using real-time PCR (Additional file 2). The results were in full agreement with the pre-determined culture status of the samples.

## Discussion

The aim of this study was to demonstrate the application of RPA, a rapid amplification technology and its utility for detection of GBS in clinical samples. The current gold standard for determination of GBS status involves swab culture for 48 h in selective media [1]. The RPA assay described in this study can be completed in 10–15 min post sample processing and offers a viable alternative for rapid near patient testing.

The gene target used for development of the RPA-GBS assay was the cfb gene. This gene is present in all GBS isolates [14] and considering the sequence homegeniety among isolates is a good target for development of an inclusive molecular test. Following an extensive *in-silico* analysis of cfb gene sequences primers and probes for the RPA-GBS assay were designed according the criteria required for RPA amplification (www.twistdx.co.uk).

An extensive performance evaluation of the assay was carried out with respect to analytical specificity and sensitivity. The inclusivity of the assay was determined by testing 17 GBS isolates, all of which were detected. The exclusivity of the assay was determined by testing a panel of 12 organisms from the Streptococcus genus. None of these related organisms gave a signal in the assay. The exclusivity of the assay was further challenged by testing a panel of 78 unrelated organisms commonly found in the gastrointestinal and genital tracts. None of these organisms produced a signal, demonstrating the specificity of the RPA-GBS assay.

The limit of detection (analytical sensitivity) of the assay was determined by testing replicates of varying concentrations of GBS DNA. This was done over three independent experiments. Hit rate analysis showed the limit of detection to be between six and 12 genome copies.

The performance of the RPA assay was compared to a previously published real-time PCR [13]. The limit of detection of the real-time PCR assay was comparable to that of the RPA assay, with both achieving a limit of detection below 12.5 genome copies., The performance of both assays when applied to clinical samples was identical. Some clinical samples which presumably had low cell counts also gave late onset times (late fluorescence signals) in RPA assays and CP values in PCR assays (data not shown) but were none the less detected, indicating that the limit of detection is in the acceptable range for application to clinical samples.

From the results presented here, the potential of RPA as a technology for use in a point of care (POC) or near patient setting is clear. Furthermore, the RPA reagents can be lyophilised and because the reaction is isothermal, instrumentation requirements are relatively straightforward. The reaction time is within the 10–15 min range, which offers the significant advantage of fast turnaround time compared to many other amplification technologies. These characteristics of RPA technology mean that it would be ideally suited for the development of POC assays. Detection of GBS during labour is one application particularly suited to a point of care setting. Results are often required rapidly and the current gold standard culture method takes 48 h to turn around meaning that it is cannot be used in a labour ward setting. Even in relation to other standard molecular tests such as real-time PCR the results obtained with RPA are very promising.

In order to further optimise the RPA assay described here for full clinical validation, some additional development work would be required including optimisation of an appropriate sample preparation method, compatible with the RPA reaction, and the addition of either a process control or internal amplification control (IAC) to the assay as a target. IACs used in RPA assays have been previously reported [12].

## Conclusion

We report here the evaluation of a specific, sensitive and rapid RPA assay for GBS detection in clinical samples. The newly developed assay is specific for the target of interest and has a limit of detection between six and 12 genome copies. The assay was compared to a previously published real-time PCR assay for GBS. When applied to testing of clinical samples the performance of the RPA assay was identical to the real-time PCR assay with the added advantage that the assay time was reduced significantly to between 10 and 15 min. The newly described RPA assay could potentially be utilised in a near-patient testing setting, allowing rapid clinical decisions to be made on the necessity to administer appropriate antibiotic treatment.

## Additional files

Additional file 1: Non-streptococcal species found at the site of infection used in RPA assay exclusivity testing. (DOCX 22 kb) Additional file 2: Clinical sample analysis results. (DOCX 24 kb)

## Abbreviations

CP: The C_p_ (crossing point-PCR-cycle) value is the cycle at which fluorescence achieves a defined threshold. It corresponds to the cycle at which a statistically significant increase in fluorescence is first detected
GBS: Group B streptococcus
PCR: Polymerase chain reaction
RPA: Recombinase polymerase amplification

## References

[1] Verani JR, Mcgee L, Schrag SJ (2010). Prevention of Perinatal Group B Streptococcal Disease.

[2] Oh W (2013). Early onset neonatal group B streptococcal sepsis. Am J Perinatol.

[3] Hughes RG, Brocklehurst P, Heath P, Stenson B (2012). Prevention of Early-onset Neonatal Group B Streptococcal Disease.

[4] Schuchat A (1999). Group B streptococcus. Lancet.

[5] Schrag SJ, Zywicki S, Farley MM, Reingold AL, Harrison LH, Lefkowitz LB, Hadler JL, Danila R, Cieslak PR, Schuchat A (2000). Group B streptococcal disease in the era of intrapartum antibiotic prophylaxis. N Engl J Med.

[6] Schrag SJ, Smith G, Gamble M, Schuchat A, Zell ER, Lynfield R, Roome A, Arnold KE, Craig AS, Harrison LH, Reingold A, Stefonek K, Active Bacterial Core, S (2002). A population-based comparison of strategies to prevent early-onset group B streptococcal disease in neonates. N Engl J Med.

[7] World Health Organisation (2005). State of the art of vaccine research and development.

[8] Schuchat A (1998). Epidemiology of group B streptococcal disease in the United States: shifting paradigms. Clin Microbiol Rev.

[9] Larsen JW, Sever JL (2008). Group B streptococcus and pregnancy: a review. Am J Obstet Gynecol.

[10] Berardi A, Perrone E, Ciccia M, Tridapalli E, Piepoli M, Contiero R, Ferrari F, Rossi C, Lugli L, Creti R, Bacchi Reggiani ML, Lanari M, Memo L, Pedna MF, Venturelli C (2013). Group B streptococcus late-onset disease: 2003–2010. Pediatrics.

[11] Piepenburg O, Williams CH, Stemple DL, Armes NA (2006). DNA detection using recombination proteins. PLoS Biol.

[12] Daher RK, Stewart G, Boissinot M, Bergeron MG (2014). Isothermal recombinase polymerase amplification assay applied to the detection of Group B streptococci in vaginal/anal samples. Clin Chem.

[13] Wernecke M, Mullen C, Sharma V, Morrison J, Barry T, Maher M, Smith TJ (2009). Evaluation of a novel real-time PCR test based on the ssrA gene for the identification of group B streptococci in vaginal swabs. BMC Infect Dis.

[14] Gosiewski T, Brzychczy-Wloch M, Heczko PB (2012). The application of multiplex PCR to detect seven different DNA targets in group B streptococci. Folia Microbiol.

