# Supplementary material for: Development and performance evaluation of a recombinase polymerase amplification assay for the rapid detection of group B streptococcus
Source: BMC Microbiol. 2016 Sep 22;16:221. doi: 10.1186/s12866-016-0836-y (PMC5034592; doi:10.1186/s12866-016-0836-y)
Supplement: Additional file 1: — Non-streptococcal species found at the site of infection used in RPA assay exclusivity testing. (DOCX 22 kb) [file 12866_2016_836_MOESM1_ESM.docx]

**File Name:** Additional File 1

**Title of Data:** Non-Streptococcal species found at the site of infection used in RPA assay exclusivity testing.

**Description of Data:** Bacterial, fungal and parasitic organisms commonly found at the site of infection which were used for specificity testing the GBS RPA assay.

| Organism | Source | ID | Result |
| --- | --- | --- | --- |
| *Acinetobacter baumanii* | DSMZ | 30007 | Not Detected |
| *Acinetobacter calcoaceticus* | ATCC | 51432 | Not Detected |
| *Acinetobacter iwoffii* | DSMZ | 2403 | Not Detected |
| *Alcaligenes faecalis* | DSMZ | 13644 | Not Detected |
| *Alcaligenes faecalis subsp. faecalis* | DSMZ | 30030 | Not Detected |
| *Anaerococcus vaginalis* | DSMZ | 7457 | Not Detected |
| *Atopobium vaginae* | DSMZ | 15829 | Not Detected |
| *Bacteroides bivius* | DSMZ | 20514 | Not Detected |
| *Bacteroides disiens* | DSMZ | 20516 | Not Detected |
| *Bacteroides fragilis* | DSMZ | 2151 | Not Detected |
| *Bacteroides melaninogenica* | DSMZ | 7089 | Not Detected |
| *Bacteroides oralis* | DSMZ | 20702 | Not Detected |
| *Bacteroides ovatus* | DSMZ | 1896 | Not Detected |
| *Bacteroides thetaiotaomicron* | DSMZ | 2079 | Not Detected |
| *Bacteroides uniformis* | DSMZ | 6597 | Not Detected |
| *Bacteroides vulgatus* | DSMZ | 1447 | Not Detected |
| *Bifidobacterium bifidum* | DSMZ | 20456 | Not Detected |
| *Bifidobacterium breve* | DSMZ | 20213 | Not Detected |
| *Bifidobacterium dentium* | DSMZ | 20436 | Not Detected |
| *Campylobacter concisus* | DSMZ | 9716 | Not Detected |
| *Campylobacter hominis* | DSMZ | 21671 | Not Detected |
| *Campylobacter jejuni* | DSMZ | 4688 | Not Detected |
| *Citrobacter freundii* | ATCC | 8090 | Not Detected |
| *Corynebacterium genitalium* | NCTC | 12451 | Not Detected |
| *Corynebacterium genitalium* | NCTC | 11859 | Not Detected |
| *Corynebacterium sundsvallense* | DSMZ | 44613 | Not Detected |
| *Enterobacter cloacae* | ATCC | 13047 | Not Detected |
| *Enterococcus faecalis* | DSMZ | 20371 | Not Detected |
| *Enterococcus faecium* | DSMZ | 20477 | Not Detected |
| *Escherichia coli* | DSMZ | 30083 | Not Detected |
| *Fusobacterium gonidiaformans* | DSMZ | 19810 | Not Detected |
| *Fusobacterium nucleatum* | DSMZ | 15643 | Not Detected |
| *Fusobacterium varium* | DSMZ | 19868 | Not Detected |
| *Klebsiella pneumoniae* | DSMZ | 30184 | Not Detected |
| *Lactobacillus acidophilus* | DSMZ | 20079 | Not Detected |
| *Lactobacillus brevis* | DSMZ | 20054 | Not Detected |
| *Lactobacillus crisptus* | DSMZ | 20584 | Not Detected |
| *Lactobacillus fermentum* | DSMZ | 20055 | Not Detected |
| *Lactobacillus gasseri* | DSMZ | 20243 | Not Detected |
| *Lactobacillus iners* | DSMZ | 13335 | Not Detected |
| *Lactobacillus jensenii* | DSMZ | 20557 | Not Detected |
| *Lactobacillus lactis lactis* | DSMZ | 20072 | Not Detected |
| *Lactobacillus oris* | DSMZ | 4864 | Not Detected |
| *Lactobacillus vaginalis* | DSMZ | 5837 | Not Detected |
| *Lactococcus lactis cremoris* | DSMZ | 20069 | Not Detected |
| *Leptotrichia amnionii* | DSMZ | 16630 | Not Detected |
| *Mobiluncus curtisii* | DSMZ | 2711 | Not Detected |
| *Mobiluncus mulieris* | DSMZ | 2710 | Not Detected |
| *Moraxella (Moraxella) osloensis* | DSMZ | 6998 | Not Detected |
| *Moraxella catarrhalis* | DSMZ | 11994 | Not Detected |
| *Morganella morganii* | DSMZ | 30164 | Not Detected |
| *Mycoplasma hominis* | NCTC | 10111 | Not Detected |
| *Peptococcus magnus* | DSMZ | 20470 | Not Detected |
| *Peptococcus niger* | DSMZ | 20475 | Not Detected |
| *Peptostreptococcus anaerobius* | DSMZ | 2949 | Not Detected |
| *Peptostreptococcus parvulus (Atopobium parvulum)* | DSMZ | 20469 | Not Detected |
| *Peptostreptococcus productus* | DSMZ | 2950 | Not Detected |
| *Porphyromonas asaccharolytica* | DSMZ | 20707 | Not Detected |
| *Prevotella bivia* | DSMZ | 20514 | Not Detected |
| *Prevotella corporis* | DSMZ | 18810 | Not Detected |
| *Propionibacterium acnes* | DSMZ | 1897 | Not Detected |
| *Proteus mirabilis* | DSMZ | 4479 | Not Detected |
| *Proteus vulgaris* | DSMZ | 2140 | Not Detected |
| *Providencia stuartii* | DSMZ | 4539 | Not Detected |
| *Pseudomonas aeruginosa* | DSMZ | 50071 | Not Detected |
| *Pseudomonas putida* | DSMZ | 291 | Not Detected |
| *Serratia marcescens* | DSMZ | 1608 | Not Detected |
| *Staphylococcus aureus* | DSMZ | 346 | Not Detected |
| *Staphylococcus epidermidis* | DSMZ | 20044 | Not Detected |
| *Staphylococcus intermedius* | DSMZ | 20373 | Not Detected |
| *Streptococcus milleri* | DSMZ | 20386 | Not Detected |
| *Ureaplasma urealyticum* | ATCC | 27619 | Not Detected |
| *Veillonella parvula* | DSMZ | 2008 | Not Detected |
| *Veillonella parvula subsp. Atypica* | DSMZ | 20739 | Not Detected |
| *Candida albicans* | CBS | 7200 | Not Detected |
| *Gardnerella vaginalis* | CCUG | 3717 | Not Detected |
| *Trichomonas vaginalis* | ATCC | 30093 | Not Detected |
| *Neisseria gonorrhoeae* | DSMZ | 9188 | Not Detected |

### ATTC American Tissue Culture Collection

### NCTC National Collection of Type Cultures

### CBS Centraalbureau Schimmelcultures

### CCUG Culture Collection of the University of Gothenburg
