# Supplementary material for: Development and performance evaluation of a recombinase polymerase amplification assay for the rapid detection of group B streptococcus
Source: BMC Microbiol. 2016 Sep 22;16:221. doi: 10.1186/s12866-016-0836-y (PMC5034592; doi:10.1186/s12866-016-0836-y)
Supplement: Additional file 2: — Clinical sample analysis results. (DOCX 24 kb) [file 12866_2016_836_MOESM2_ESM.docx]

File Name: Additional File 2

Title of Data: Clinical Sample Analysis Results

**Description of Data:** Comparison of real-time PCR, RPA and culture results obtained for clinical samples.

|  | **Clinical Sample No.** | **Real-time PCR** | **RPA Assay** | **GBS Culture Status** |
| --- | --- | --- | --- | --- |
| 1 | 1319 | Positive | Positive | Positive |
| 2 | 1320 | Positive | Positive | Positive |
| 3 | 1324 | Positive | Positive | Positive |
| 4 | 1326 | Positive | Positive | Positive |
| 5 | 1327 | Positive | Positive | Positive |
| 6 | 1328 | Positive | Positive | Positive |
| 7 | 1329 | Positive | Positive | Positive |
| 8 | 1330 | Positive | Positive | Positive |
| 9 | 1331 | Positive | Positive | Positive |
| 10 | 1332 | Positive | Positive | Positive |
| 11 | 1333 | Positive | Positive | Positive |
| 12 | 1334 | Positive | Positive | Positive |
| 13 | 1335 | Positive | Positive | Positive |
| 14 | 1337 | Positive | Positive | Positive |
| 15 | 1338 | Positive | Positive | Positive |
| 16 | 2274 | Positive | Positive | Positive |
| 17 | 2275 | Positive | Positive | Positive |
| 18 | 2277 | Positive | Positive | Positive |
| 19 | 2279 | Positive | Positive | Positive |
| 20 | 2280 | Positive | Positive | Positive |
| 21 | 2281 | Positive | Positive | Positive |
| 22 | 2287 | Positive | Positive | Positive |
| 23 | 2288 | Negative | Negative | Negative |
| 24 | 2289 | Negative | Negative | Negative |
| 25 | 2370 | Negative | Negative | Negative |
| 26 | 2373 | Negative | Negative | Negative |
| 27 | 2374 | Positive | Positive | Positive |
| 28 | 2376 | Positive | Positive | Positive |
| 29 | 2377 | Negative | Negative | Negative |
| 30 | 2379 | Positive | Positive | Positive |
| 31 | 2382 | Negative | Negative | Negative |
| 32 | 2383 | Negative | Negative | Negative |
| 33 | 2527 | Positive | Positive | Positive |
| 34 | 2528 | Positive | Positive | Positive |
| 35 | 2588 | Positive | Positive | Positive |
| 36 | 2589 | Positive | Positive | Positive |
| 37 | 2590 | Positive | Positive | Positive |
| 38 | 2763 | Positive | Positive | Positive |
| 39 | 2775 | Positive | Positive | Positive |
| 40 | 2942 | Positive | Positive | Positive |
| 41 | 2945 | Positive | Positive | Positive |
| 42 | 2946 | Positive | Positive | Positive |
| 43 | 1321 | Positive | Positive | Positive |
| 44 | 1322 | Positive | Positive | Positive |
| 45 | 2276 | Positive | Positive | Positive |
| 46 | 2278 | Positive | Positive | Positive |
| 47 | 2283 | Positive | Positive | Positive |
| 48 | 2284 | Positive | Positive | Positive |
| 49 | 2285 | Positive | Positive | Positive |
| 50 | 2367 | Positive | Positive | Positive |
| 51 | 2371 | Positive | Positive | Positive |
| 52 | 2380 | Positive | Positive | Positive |
| 53 | 2381 | Positive | Positive | Positive |
| 54 | 2555 | Positive | Positive | Positive |
| 55 | 2585 | Positive | Positive | Positive |
| 56 | 2586 | Positive | Positive | Positive |
| 57 | 2587 | Positive | Positive | Positive |
| 58 | 2591 | Positive | Uncertain | Positive |
| 59 | 2944 | Positive | Positive | Positive |
| 60 | 2947 | Positive | Positive | Positive |
| 61 | 2948 | Positive | Positive | Positive |
| 62 | 2949 | Positive | Positive | Positive |
| 63 | 2950 | Positive | Positive | Positive |
| 64 | 3016 | Positive | Positive | Positive |
| 65 | 3017 | Positive | Positive | Positive |
| 66 | 3095 | Positive | Positive | Positive |
| 67 | 3096 | Positive | Positive | Positive |
| 68 | 3097 | Positive | Positive | Positive |
| 69 | 3222 | Positive | Positive | Positive |
| 70 | 3223 | Positive | Positive | Positive |
| 71 | 3264 | Positive | Positive | Positive |
| 72 | 3265 | Positive | Positive | Positive |
| 73 | 3266 | Positive | Positive | Positive |
| 74 | 3267 | Positive | Positive | Positive |
| 75 | 3268 | Positive | Positive | Positive |
| 76 | 3273 | Positive | Positive | Positive |
| 77 | 3274 | Positive | Positive | Positive |
| 78 | 3281 | Positive | Positive | Positive |
| 79 | 3282 | Positive | Positive | Positive |
| 80 | 3283 | Positive | Positive | Positive |
| 81 | 3288 | Positive | Positive | Positive |
| 82 | 3289 | Positive | Positive | Positive |
| 83 | 3290 | Positive | Positive | Positive |
| 84 | 3512 | Positive | Positive | Positive |
| 85 | 3513 | Positive | Positive | Positive |
| 86 | 3514 | Positive | Positive | Positive |
| 87 | 3515 | Positive | Positive | Positive |
| 88 | 3521 | Positive | Positive | Positive |
| 89 | 3638 | Positive | Positive | Positive |
| 90 | 3639 | Positive | Positive | Positive |
| 91 | 5 | Negative | Negative | Negative |
| 92 | 6 | Negative | Negative | Negative |
| 93 | 7 | Negative | Negative | Negative |
| 94 | 9 | Negative | Negative | Negative |
| 95 | 10 | Positive | Positive | Positive |
| 96 | 12 | Negative | Negative | Negative |
| 97 | 13 | Negative | Negative | Negative |
| 98 | 14 | Negative | Negative | Negative |
| 99 | 15 | Negative | Negative | Negative |
| 100 | 20 | Positive | Positive | Positive |
| 101 | 21 | Negative | Negative | Negative |
| 102 | 22 | Negative | Negative | Negative |
| 103 | 23 | Negative | Negative | Negative |
| 104 | 24 | Negative | Negative | Negative |
| 105 | 25 | Negative | Negative | Negative |
| 106 | 2368 | Negative | Negative | Negative |
| 107 | 2369 | Negative | Negative | Negative |
| 108 | 16 | Negative | Negative | Negative |
| 109 | 17 | Negative | Negative | Negative |
| 110 | 18 | Negative | Negative | Negative |
| 111 | 19 | Negative | Negative | Negative |
| 112 | 26 | Negative | Negative | Negative |
| 113 | 27 | Negative | Negative | Negative |
| 114 | 28 | Negative | Negative | Negative |
| 115 | 29 | Negative | Negative | Negative |
| 116 | 30 | Negative | Negative | Negative |
| 117 | 31 | Negative | Negative | Negative |
| 118 | 32 | Negative | Negative | Negative |
| 119 | 33 | Negative | Negative | Negative |
| 120 | 34 | Negative | Negative | Negative |
| 121 | 35 | Negative | Negative | Negative |
| 122 | 36 | Negative | Negative | Negative |
| 123 | 37 | Negative | Negative | Negative |
| 124 | 39 | Negative | Negative | Negative |
